# A Case of Glycogenic Hepatopathy as a Complication of Poorly Controlled Type 1 Diabetes Mellitus

**DOI:** 10.1155/2022/8939867

**Published:** 2022-09-29

**Authors:** Samhitha Munugoti, Vamsee Reddy, Gaurav Patel, Maneesh Gaddam, Triveni Abburi

**Affiliations:** ^1^Department of Internal Medicine, Prime Health Care Consortium at St. Mary's and St. Clare Denville Hospital Program, Denville, NJ, USA; ^2^Division of Pulmonary, Critical Care, and Sleep Medicine, Tufts Medical Center, Boston, MA, USA

## Abstract

A 23-year-old African American male with a medical history significant for poorly controlled type 1 diabetes mellitus (T1DM) presented with abdominal pain and vomiting. His laboratory workup was consistent with diabetic ketoacidosis (DKA). An acute elevation of liver enzymes was noted as the DKA resolved, with the alanine transferase and aspartate transferase levels elevated to more than 50 times the normal limit within the next 24 hours. Because abnormal liver function tests are found frequently in patients with type 1 diabetes mellitus, it is important to have a broad differential diagnosis. Furthermore, a low threshold of suspicion is required to identify a relatively underdiagnosed etiology like glycogenic hepatopathy (GH). This case report describes how patterns and trends of liver function tests provide important clues to the diagnosis of GH; how imaging modalities like ultrasonography, computerized tomography (CT) scan, and magnetic resonance imaging (MRI) scan could be used to differentiate GH from nonalcoholic fatty liver disease (NAFLD); and how the diagnosis of GH can be made without the need for invasive liver biopsy. The knowledge about GH should prevent its delayed diagnosis and improve the outcomes by appropriately managing uncontrolled type 1 DM.

## 1. Introduction

Patients with type 1 diabetes are frequently associated with abnormal liver function tests. Therefore, further investigation is often required to evaluate for associated hepatic abnormalities such as abnormal glycogen deposition, nonalcoholic fatty liver disease (NAFLD), fibrosis, and cirrhosis [1]. The most frequent etiology of elevated aminotransferases in type 1 diabetes is NAFLD [2]. However, a rare, underdiagnosed but important cause of liver injury is glycogenic hepatopathy (GH), first documented as a component of Mauriac syndrome in 1930 [3]. Since then, many cases due to other etiologies have been reported. These include poorly controlled diabetes mellitus, dumping syndrome after gastrectomy, anorexia nervosa, use of high-dose corticosteroids, and high doses of insulin [4–7]. While GH has been traditionally diagnosed by liver biopsy, less invasive alternatives have been suggested [8]. Improvement of hyperglycemia and hepatomegaly is commonly noted with GH. Diagnosis can be made based on clinical and radiological data, and liver biopsy becomes essential when other hepatic disorders needed to be ruled out.

## 2. Case Presentation

A 23-year-old African American male with type I diabetes mellitus diagnosed at the age of 3 presented with abdominal pain and vomiting for two days. His home insulin regimen included 40 units/day of regular insulin in divided doses along with insulin lispro with meals, the dosage of which he was not aware. The patient did not recall when he took his last dose of insulin but reported frequent episodes of high glucose levels previously. His family members confirmed his noncompliance to the insulin regimen, primarily due to a lack of understanding of his medical condition and poor socioeconomic status.

On presentation, the patient was hemodynamically stable and afebrile along with normal heart and respiratory rates. He was of tall, thin build with a BMI of 19. He was alert and awake with an unremarkable cardiac and respiratory examination. Abdominal examination revealed no guarding, rigidity, or tenderness but was noted to have hepatomegaly.

On initial laboratory studies, his complete blood count was unremarkable. The basic metabolic profile revealed sodium of 132 mEq/L, potassium of 5.3 mEq/L, chloride of 89 mEq/L, carbon dioxide of 14 mEq/L, blood urea nitrogen of 19.4 mg/dL, creatinine of 1.3 mg/dL, and an anion gap of 29 mEq/L. His glucose levels were found to be 566 mg/dL. Arterial blood gas showed a pH of 7.3, pCO2 of 24 mm of Hg, pO2 of 107 mm of Hg, HCO3 of 11.7 mmol/L, and lactate of 2.4 mmol/L. He was noted to have positive acetones and ketones. This was consistent with diabetic ketoacidosis in the setting of poor compliance, with the Hba1c levels of 17%.

The patient was initially given 8 units of regular insulin and was started on an insulin drip at 0.1 U/Kg. Over the next 12 hours, the patient's DKA resolved and he was transitioned to subcutaneous insulin. Liver function tests at that time revealed aspartate aminotransferase (AST) of 816 U/L, alanine aminotransferase (ALT) of 569 U/L, and alkaline phosphatase (ALP) of 132 U/L. An extensive workup for elevated aminotransferases was pursued. Further history was obtained with no recent travel, acetaminophen ingestion, and any other substances including alcohol, herbal, or over-the-counter medications. There was no family history or past medical history for any liver disease. Drug screens for salicylates, acetaminophen, amphetamines, barbiturates, benzodiazepines, cocaine, ethanol, and cannabinoids were negative. Serology was negative for viral hepatitis A, B, and C. Antismooth muscle antibodies were negative ruling out autoimmune hepatitis. A normal ceruloplasmin level of 28 mg/dL with no Kayser–Fleischer rings on examination, no neurological or psychiatric manifestations, or family history of Wilson's disease made the diagnosis of Wilson's disease unlikely.

**Figure 1:**
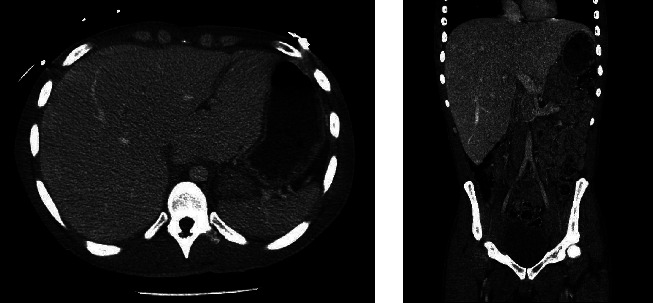
Axial and coronal sections of the abdominal CT scan showing hepatomegaly with a hyperdense liver.

**Figure 2:**
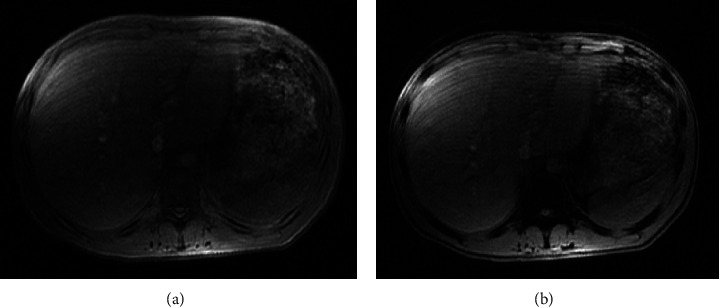
T1-MRI images showing no difference in the signal intensities between the in-phase (a) and opposed-phase (b).

Over the next 24 hours, his liver enzymes continued to increase dramatically with AST of 10,499 U/L, ALT of 2,486 U/L, and ALP of 230 U/L. The synthetic liver function tests which included serum albumin, prothrombin time, and international normalized ratio were within normal limits. Extreme elevations of aminotransferases can be precipitated by ischemic hepatopathy. However, there were no episodes of hypotension, and lactic acid was mildly elevated to 2.4 mmol/L during his hospitalization. Ultrasonography of the abdomen was performed which was consistent with hepatomegaly but no steatosis or cirrhosis. Nonalcoholic fatty liver disease (NAFLD) and glycogenic hepatopathy (GH) were the two major considerations at this point. A CT scan of the abdomen/pelvis revealed hyperdense hepatomegaly and the density was greater than the spleen (Figure 1). The ratio of liver-to-spleen (L/S) Hounsfield units (HU) was 1.56. Although pointing towards GH and against NAFLD, an MRI scan was performed for hepatomegaly. On this imaging, there was no significant difference in liver intensity between the in-phase and opposed-phase sequences, confirming the diagnosis of GH (Figure 2). Although liver biopsy is considered the gold standard for diagnosing GH, there was enough clinical and radiological evidence to diagnose the patient with GH.

**Figure 3:**
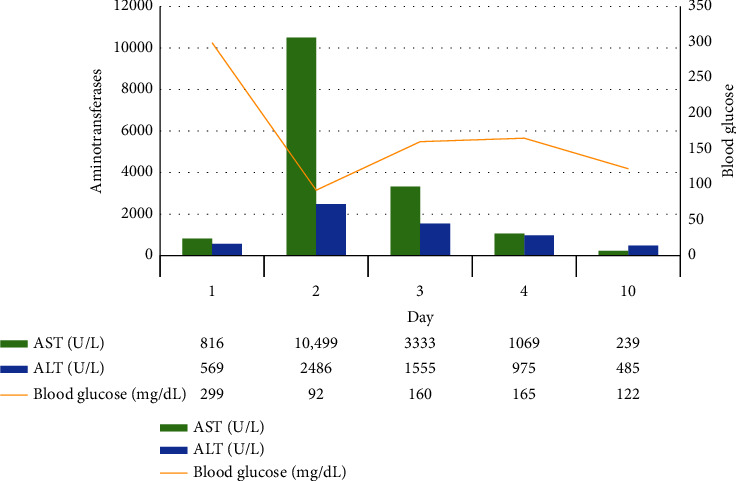
Relationship between the trends of aminotransferases and blood glucose levels. AST: aspartate aminotransferase; ALT: alanine aminotransferase.

The patient's aminotransferases started trending down as the blood glucose levels were better controlled during the admission with 17 units of insulin glargine once daily and 6-8 units of insulin lispro premeal (Figure 3). A follow-up after 10 days confirmed improving aminotransferases with well-controlled blood sugar levels.

## 3. Discussion

Patients who present with frequent episodes of DKA and treated with high doses of insulin may often have GH. However, the incidence is unknown as GH is relatively underdiagnosed and therefore underestimated. The presentation is nonspecific and can range from abdominal pain, nausea, and vomiting to an asymptomatic elevation of aminotransferases [9]. Hepatomegaly is the most common finding noted on physical examination [9].

GH is caused by a reversible accumulation of excess glycogen in the liver. This excessive accumulation in the liver causes hepatomegaly and hepatocyte injury leading to elevated liver enzymes [10–12]. Glycogenesis and glycogenolysis are the two processes occurring in the liver to balance the glycogen, which is the reservoir of glucose. Elevated serum glucose levels in poorly controlled diabetes lead to the diffusion of glucose into the hepatocytes facilitated through the GLUT 2 transporter, which occurs independently of insulin [13]. The glucose is then trapped in the hepatocytes after irreversible phosphorylation by the enzyme glucokinase to glucose-6-phosphate. Upon initiating treatment with insulin, the glucose-6-phosphate is now polymerized to glycogen by the enzyme glycogen synthase, which continues for some time despite a drop in insulin levels [13, 14]. This continued accumulation of glycogen leads to hepatomegaly. This acute presentation of hepatomegaly occurs when prolonged or marked hyperglycemia is treated with exogenous insulin [15, 16]. Therefore, it is only obvious that the description of Mauriac syndrome in 1930 happened only shortly after the availability of insulin treatment [17].

Given the rarity of this condition, a high index of suspicion is required to diagnose GH. The diagnosis should be suspected when elevated liver enzymes are noted in a patient with uncontrolled T1DM, especially with exaggerated hemoglobin A1c levels [18]. The pattern of the elevated liver enzymes provides important clues to differentiate the various hepatic abnormalities. GH is associated with the following patterns of abnormal liver enzymes:

1. Elevations in ALT and AST in out of proportion to ALP and bilirubin indicating predominant hepatocellular injury [13, 16, 18].
2. AST is higher than ALT [19].
3. Synthetic function of the liver is preserved, that is, normal albumin and INR levels [13, 20, 21].

GH is usually noted to have aminotransferases elevated about 13 times the upper normal limit [19]. Our patient had a pattern of liver enzymes similar to that described above. Although ALT (which is present in the highest concentration in the liver) is more specific for liver injury than AST (which is found in the liver, cardiac muscle, skeletal muscle, kidneys, brain, pancreas, lungs, leukocytes, and erythrocytes), there are few other liver conditions where the AST is higher than ALT. Common etiologies include alcoholic liver disease, Wilson's disease, cirrhosis due to viral hepatitis, and nonalcoholic fatty liver disease (NAFLD). While NAFLD is more commonly observed in obese patients with type 2 DM, GH is more common in patients with poorly controlled type 1 DM. However, the diagnostic dilemma continues as distinguishing GH from NAFLD is extremely difficult purely based on clinical presentation and laboratory work.

Various imaging modalities can be used to support or disprove the diagnosis of GH. Although occasionally normal, the hepatic ultrasonography of GH would reveal hepatomegaly with uniform echogenicity due to glycogen accumulation. While NAFLD occurs due to fat deposition in the liver, the findings of ultrasonography of the hepatic tissues of NAFLD are similar to those of GH. The ultrasound imaging in our patient revealed hepatomegaly with no evidence of steatosis and cirrhosis. Therefore, ultrasound is not a useful imaging modality to distinguish GH and NAFLD [9, 22].

An abdominal CT can provide clues to diagnose GH. The liver density on the abdominal CT helps differentiate GH from NAFLD. The glycogen accumulation in GH gives a hyperdense appearance to the liver while the fatty deposition gives a hypodense appearance in NAFLD. The difference in the liver density as a clue to GH has been reported by Sweetser and Kraichely [23]. In addition, Doppman et al. reported a dose-dependent increase in the liver density as the glycogen content increases and a decrease in the liver density as the fat content increases [24]. The CT imaging in our patient does reveal hyperdensity of the liver with an L/S HU ratio of >1, pointing against NAFLD [25]. However, further workup is indeed warranted to confirm the diagnosis of GH.

MRI scan provides a clear distinction between GH and NAFLD. T1-MRI is recorded with in-phase and opposed-phase conditions. Intrahepatic fat storage in NAFLD causes a significantly higher signal intensity in the in-phase than in the opposed-phase [23, 26]. However, no difference in the signal intensities between the two phases is noted in GH [8, 26]. Similar MRI findings were noted in our patient with no difference in the signal intensities between the in-phase and opposed-phased phases.

Liver biopsy is considered the gold standard for diagnosing GH. Histologically, GH reveals pale, swollen hepatocytes due to marked glycogen accumulation, and intact architecture with no significant fibrosis, necrosis, inflammation, or fatty change [8]. A hematoxylin and eosin stain of the biopsy specimen would show swollen hepatocytes with prominent plasma membranes, increased volume of cytoplasm, and numerous glycogenated nuclei [9]. Staining with Periodic-Acid Schiff (PAS) shows abundant cytoplasmic glycogen deposits, which disappear after the addition of diastase that causes an enzymatic breakdown of glycogen in the hepatocytes [16, 21].

While the liver biopsy is considered the gold standard, given the invasiveness of the intervention, it is recommended that intensive insulin therapy is tried before proceeding with the liver biopsy [9]. In addition, combining a gradient dual-echo MRI sequence with a thorough clinical evaluation, biochemical workup and other radiological modalities could confirm the diagnosis of GH [8]. Our patient was diagnosed with GH based on the constellation of imaging findings and ruling out other etiologies, without having to proceed with the invasive intervention of liver biopsy.

Improving glycemic control is considered the mainstay of treatment for GH, which usually results in clinical and biochemical amelioration in 2-14 weeks [27]. Repeated episodes of DKA could cause recurrence of GH; therefore, compliance with glycemic control stays as the main therapeutic goal [18].

## 4. Conclusion

GH is an under-recognized condition and physicians should consider it in the differential diagnoses of patients with uncontrolled T1DM presenting with elevated aminotransferases. Although considered the gold standard, a liver biopsy is not always necessary and GH can be considered a diagnosis of exclusion in the right clinical setting by ruling out other etiologies combined with imaging modalities.

## Data Availability

Data sharing is not applicable to this article as no datasets were generated or analyzed during the current study.

## References

[1] Mohamed J., Nazratun Nafizah A. H., Zariyantey A. H., Budin S. B. (2016). Mechanisms of diabetes-induced liver damage: the role of oxidative stress and inflammation. *Sultan Qaboos University Medical Journal*.

[2] Angulo P. (2002). Nonalcoholic fatty liver disease. *New England Journal of Medicine*.

[3] Mauriac P. (1930). Gros ventre, hepatomegalie, troubles de las croissance chez les enfants diabetiques traits depuis plusieurs annes par l’insuline. *Gax Hebd Med Bordeaux*.

[4] Resnick J. M., Zador I., Fish D. L. (2011). Dumping syndrome, a cause of acquired glycogenic hepatopathy. *Pediatric and Developmental Pathology*.

[5] Kransdorf L. N., Millstine D., Smith M. L., Aqel B. A. (2016). Hepatic glycogen deposition in a patient with anorexia nervosa and persistently abnormal transaminase levels. *Clinics and Research in Hepatology and Gastroenterology*.

[6] Iancu T. C., Shiloh H., Dembo L. (1986). Hepatomegaly following short-term high-dose steroid therapy. *Journal of Pediatric Gastroenterology and Nutrition*.

[7] Tsujimoto T., Takano M., Nishiofuku M. (2006). Rapid onset of glycogen storage hepatomegaly in a type-2 diabetic patient after a massive dose of long-acting insulin and large doses of glucose. *Internal Medicine*.

[8] Murata F., Horie I., Ando T. (2012). A case of glycogenic hepatopathy developed in a patient with new-onset fulminant type 1 diabetes: the role of image modalities in diagnosing hepatic glycogen deposition including gradient-dual-echo MRI. *Endocrine Journal*.

[9] Sherigar J. M., Castro J. de, Yin Y. M., Guss D., Mohanty S. R. (2018). Glycogenic hepatopathy: a narrative review. *World Journal of Hepatology*.

[10] Carcione L., Lombardo F., Messina M. F., Rosano M., de Luca F. (2003). Liver glycogenosis as early manifestation in type 1 diabetes mellitus. *Diabetes, Nutrition & Metabolism*.

[11] Torres M., López D. (2001). Liver glycogen storage associated with uncontrolled type 1 diabetes mellitus. *Journal of Hepatology*.

[12] Abaci A., Bekem O., Unuvar T. (2008). Hepatic glycogenosis: a rare cause of hepatomegaly in type 1 diabetes mellitus. *Journal of Diabetes and its Complications*.

[13] Chatila R., West B. A. (1996). Hepatomegaly and abnormal liver tests due to glycogenosis in adults with diabetes. *Medicine*.

[14] Ferrer J. C., Favre C., Gomis R. R. (2003). Control of glycogen deposition. *FEBS Letters*.

[15] Stone B. G., van Thiel D. H. (1985). Diabetes mellitus and the liver. *Seminars in Liver Disease*.

[16] Torbenson M., Chen Y. Y., Brunt E. (2006). Glycogenic hepatopathy: an underrecognized hepatic complication of diabetes mellitus. *The American Journal of Surgical Pathology*.

[17] Brar D. (2009). The history of insulin. http://www.med.uni-giessen.de/itr/history/inshist.html.

[18] Messeri S., Messerini L., Vizzutti F., Laffi G., Marra F. (2012). Glycogenic hepatopathy associated with type 1 diabetes mellitus as a cause of recurrent liver damage. *Annals of Hepatology*.

[19] Haffar S., Izzy M., Habib H. (2021). Liver chemistries in glycogenic hepatopathy associated with type 1 diabetes mellitus: a systematic review and pooled analysis. *Liver International*.

[20] Hudacko R. M., Manoukian A. v, Schneider S. H., Fyfe B. (2008). Clinical resolution of glycogenic hepatopathy following improved glycemic control. *Journal of Diabetes and its Complications*.

[21] Saadi T. (2012). Glycogenic hepatopathy: a rare disease that can appear and resolve rapidly in parallel with glycemic control. *The Israel Medical Association Journal*.

[22] Khoury J., Zohar Y., Shehadeh N., Saadi T. (2018). Glycogenic hepatopathy. *Hepatobiliary and Pancreatic Diseases International*.

[23] Sweetser S., Kraichely R. E. (2010). The bright liver of glycogenic hepatopathy. *Hepatology*.

[24] Doppman J. L., Cornblath M., Dwyer A. J., Adams A. J., Girton M. E., Sidbury J. (1982). Computed tomography of the liver and kidneys in glycogen storage disease. *Journal of Computer Assisted Tomography*.

[25] Zeb I., Li D., Nasir K., Katz R., Larijani V. N., Budoff M. J. (2012). Computed tomography scans in the evaluation of fatty liver disease in a population based study: the multi-ethnic study of atherosclerosis. *Academic Radiology*.

[26] Saikusa M., Yatsuga S., Tonan T., Koga Y. (2013). Glycogenic hepatopathy and non-alcoholic fatty liver disease in type 1 diabetes patients. *Pediatrics International*.

[27] Cha J. H., Ra S. H., Park Y. M. (2013). Three cases of glycogenic hepatopathy mimicking acute and relapsing hepatitis in type I diabetes mellitus. *Clinical and Molecular Hepatology*.

